# Telomere dynamics in maturing frogs vary among organs

**DOI:** 10.1098/rsbl.2024.0626

**Published:** 2025-02-26

**Authors:** Pablo Burraco, Neil B. Metcalfe, Pat Monaghan

**Affiliations:** ^1^ Doñana Biological Station (CSIC), Seville 41092, Spain; ^2^ School of Biodiversity, One Health and Veterinary Medicine, College of Medical, Veterinary and Life Sciences, University of Glasgow, Glasgow G12 8QQ, UK

**Keywords:** ageing, amphibians, developmental biology, complex life cycles, life-history traits, temperature

## Abstract

It is important to know whether organs age at the same rate and are equally affected by developmental conditions as this provides insights into causes of ageing. However, data on organ-specific telomere dynamics remain scant. In a previous study of the early life of the amphibian *Xenopus laevis*, we detected changes in telomere lengths in gut cells, while liver, heart and muscle telomeres were unchanged; larval rearing temperature had minimal effects. Here, we extend that study to examine telomere dynamics in the same four organs and larval temperature treatments from 70-day post-metamorphic juvenile *Xenopus* through to sexually mature (2-year-old) adults. Telomeres shortened from juvenile to adult in the gut, heart and hindlimb muscle. In contrast, liver telomere lengths did not change with age but were shorter if the early life temperature was warm. Organ telomere lengths were influenced by sex only in adults. Warmer larval temperatures were also associated with longer gut telomeres in juveniles. Hence, pre-metamorphic conditions can influence post-metamorphic telomere dynamics, and telomere loss between juvenile and adult life stages occurs in different organs from those affected earlier in life. These findings indicate the existence of organ-dependent ageing rates across lifetimes, potentially related to developmental and environmental history.

## 1. Introduction

Understanding heterogeneity in the rate at which organs age, and how this is influenced by environmental conditions, is a major goal in evolutionary biology and biogerontology, as this may help us explain inter-individual differences in both healthspan and lifespan. However, while ageing rates are thought to differ among the body organs of an organism [1,2] and potentially be differentially susceptible to early life conditions [3–5], organ-specific data are primarily available for humans [6–8], with much less research conducted in other species (but see [9–13]).

The erosion of telomeres (the protective ends capping linear chromosomes) has been identified as a key hallmark of cellular ageing [14], and telomere dynamics have been extensively used in eco-evolutionary studies to investigate how environmental variation affects animal biological state and ageing [15–18]. Telomeres generally shorten throughout life across vertebrates, and variation within species in the pattern and pace of this shortening has been linked to lifespan and environmental factors. However, the current evidence is largely from endotherms [19–24], and most of these studies have been focused on a single organ or tissue (often blood [25]). Environmental influences on the rate of ageing are known to differ between endotherms and ectotherms. For instance, ectothermic animals are likely to be more affected by climate change [26], since warmer temperatures increase their growth rates and metabolism, which in turn is thought to influence their pace of ageing; this is reflected in changes in telomere dynamics [27]. Also, the vast majority of ectotherms undergo metamorphosis, which involves the loss, major remodelling or creation of new organs partway through life. Yet, we know almost nothing about variation in the rate of ageing of ectotherms across organs or life stages.

Knowledge of telomere dynamics is particularly lacking for amphibians, the globally most threatened vertebrate group. Thermal conditions shape amphibian larval development and often result in metabolic carry-over effects [28,29], which potentially result in telomere shortening. Most amphibians undergo a complete metamorphosis, involving abrupt tissue or organ remodelling and transformation to allow the transition from a fish-like organism to a frog. Some organs (e.g. the tail) are lost at metamorphosis, some are grown anew (e.g. the limbs), some retain the same structure (e.g. the heart and liver) and some are re-modelled (e.g. the gut). Following metamorphosis, somatic organs in amphibians do not show major developmental changes but often exhibit large changes in size. Depending on the organ, this change in size can come either by hyperplasia (cell division) or by hypertrophy (increase in cell size; [30–34]).

Only four studies have measured telomere length across amphibian life stages [9,35–37], and our previous research on clawed frogs was the only one in which organ-specific telomere dynamics were examined [9]. In that study, we measured changes in telomere length in four organs (gut, liver, heart and hindlimb muscle) from the larval (tadpole) stage through the metamorphic transition to juvenile frogs, and also taking post-metamorphic measurements from frogs at 70 days old (approx. 23 days post-metamorphosis) and segven months old (the age at which sexual maturation usually starts in this species). We found that gut telomere length increased dramatically during the period of metamorphosis and then decreased post-metamorphosis; liver telomere length decreased only between larvae and the seven months post-metamorphic stage (i.e. no measurable decline after metamorphosis), while heart and hindlimb muscle telomere lengths remained unchanged across the studied life stages [9]. While we manipulated environmental temperature during the pre-metamorphic larval period, we found that this had no significant effect on telomere dynamics in very early adulthood [8]. However, delayed effects of early life conditions can appear later in life, as has been found in other vertebrates, so changes may emerge in maturing organisms [38,39].

Following up on our previous study [9], here we compare telomere lengths in the gut, liver, heart and hindlimb muscle of early post-metamorphic 70-day-old frogs (approx. 18 days after metamorphosis, i.e. when most of remodelling has been completed) to those in 2-year-old adult frogs. We acknowledge the value of studying telomere dynamics in less-invasive tissues such as blood; however, this was not feasible for the early life stages in [9], and our focus here is on telomere dynamics in the same tissues. In [8], we included seven-month individuals, but by 2 years of age, animals are fully sexually mature and body size has reached the growth asymptote typically observed in individuals of this species under laboratory conditions [40]. Furthermore, females grow much faster than males during this period, with female body mass reaching more than twice that of males. Such a substantial sex difference in growth rate would be expected to create sex differences in telomere length, especially in organs that grow by hyperplasia. We previously found that telomeres up until the seven-months stage (when there is still no sex difference in body size) were shorter in the organs that grow by hyperplasia (e.g. gut and liver) than in those that grow by hypertrophy (e.g. heart and hindlimb muscle [9]). However, given the much greater growth of females between seven months and 2 years, we expected to find the difference in telomere length between hyperplasic and hypertrophic organs to be much greater in 2-year-old females than males. We also investigated whether any effects of early life temperature become evident in adult life and whether any such effects vary among organs.

## 2. Material and methods

### (a) Experimental set-up and sampling procedure

The African clawed frog *Xenopus laevis* is a fully aquatic species that can reach sexual maturity in approximately 7–10 months and whose lifespan in the wild is approximately 10 years [41]. Once metamorphosis is completed, animals exhibit large changes in size as they grow from, on average, 1−2 g just after metamorphosis to 55−250 g as a fully mature adult, with females being considerably larger than males [40]. This species is commonly used in biological research and its developmental biology is well characterized [41].

*Xenopus laevis* larvae from five wild-type clutches, obtained from a captive rearing facility at Portsmouth University, were raised from hatching and until metamorphosis either at 19°C (the temperature used at the original natal colony) or 23°C (considered non-stressful, as it is well within the temperature range experienced by the species in the wild) in 10 l tanks (*n* = 3 tanks per clutch at each temperature) and with a light : dark cycle of 12 : 12 h (full details of the experiment are given in [9]). Temperature influenced the duration of the larval period: individuals from the 19°C and 23°C treatment took, on average, 57 and 47 days to reach metamorphosis, respectively [9]. From the metamorphic transition onwards, all individuals were kept at 19°C to investigate whether larval developmental responses to temperature have consequences for post-metamorphic telomeres. Initial density was 18 larvae per 10 l tank. Several individuals per tank were collected across larval development for different purposes (see electronic supplementary material, figure S1 for further information on sampling and individual density throughout the experiment, also [9]), but resulting density did not differ between treatments or tanks, and food was adjusted throughout the experiment such that it was always supplied ad libitum. When individuals were 70 days old (‘juveniles’, an average of 23 days after metamorphosis), one frog per tank (*n* = 15 per temperature treatment) was randomly culled and the required organs (gut, liver, heart and hindlimb muscle) were sampled. Thereafter, individuals were maintained at a density of three frogs per 10 l tank until they were seven months old. At that point, one frog from each of the 15 tanks per temperature treatment was randomly chosen to be retained, and these selected individuals were then held in two separate 100 l tanks (one tank per temperature treatment, *n* = 15 individuals per tank; electronic supplementary material, figure S1). When frogs were 2 years old (‘adults’, sexually mature), the remaining individuals were euthanized (using MS-222 which is a humane UK Home Office approved method), weighed to the nearest 0.01 g and their body length (snout-to-vent) measured using callipers. The same four organs were weighed to the nearest 0.01 g and sampled for telomere analysis.

### (b) DNA extraction, sex determination and telomere length quantification

To ensure consistency in the telomere length measurements compared in this study, 70 day and 2 year samples were measured simultaneously using the methodology described below. We extracted genomic DNA using the PureLink DNA kit (Invitrogen), following the manufacturer’s protocol. We used liver DNA to determine the sex of each frog included in this study, using an established protocol based upon quantitative polymerase chain reaction (qPCR; [9,42,43]). For relative telomere length quantification (following MIQE guidelines; [44]), we used a qPCR procedure established across vertebrates [45] and optimized for *X. laevis* [9]. Intra-sample CV% was 0.98 and 0.62% for the telomere and single copy control gene (recombination-activating gene (RAG), designed for *X. laevis*) amplification, respectively. Efficiencies were 101.4 ± 1.0 (s.e.) and 98.5 ± 1.0 for the telomere and control gene amplification, respectively.

### (c) Statistical analyses

All analyses were conducted in R (v. 4.2.1). Relative telomere length data were log-transformed to meet parametric assumptions. To test whether telomere length varies across organs and life stages (juvenile versus adult), we first ran a full model with *relative telomere length* as the dependent variable, *organ*, *life stage, early life temperature* and their interactions as independent factors, *sex* and *body mass* as covariates and *qPCR plate* and *individual* as random factors. Since this model showed a significant interaction between *life stage* and *organ*, whereas neither *body mass* nor *sex* were significant (both *p*-values > 0.509; electronic supplementary material, table S1), we built a reduced model for each organ that included *relative telomere length* as the dependent variable, *life stage, early life temperature* and their interaction as independent factors, and the random factor *qPCR plate*. Within each life stage, we separately checked for the effect of the interaction between *organ*, *early life temperature*, *body mass* and *sex* by running a full linear model that also included *qPCR plate* and *individual* (nested within *clutch* for data on 70 days juveniles) as random factors. (Note that body mass and body size were highly correlated, so results of this and all other analyses were similar whether using body mass or body size as the covariate; only results for body mass are presented for reasons of clarity.)

To test whether organ telomere length correlated with organ mass (corrected for body mass) in adult frogs, we built models for each organ, including *relative telomere length* as the dependent variable, the relative mass of the *organ* and its interaction with *temperature* as independent factors, *body mass* as covariate and *qPCR plate* as the random factor. Finally, we investigated at each life stage the relationships between telomere lengths in different organs of the same individual through Pearson’s correlations with the help of the function *ggpairs* (*GGally* package). We applied Benjamini–Hochberg corrections of *p*-values for multiple comparisons using the function *p.adjust* (*stats* package).

## 3. Results

Survival was 73.5% for 70-day-old juveniles (from larva to sampling) and 76.6% for 2-year-old adults (from the point at which one frog from each clutch was pooled in two separate tanks, i.e. when frogs were seven months old, to sampling at 2 years old). Body mass did not vary significantly between the sexes in 70-day-old juveniles (2.21 ± s.e. 0.23 g, *n* = 17, for females; and 2.71 ± 0.26 g, *n* = 13, for males) but, as expected, at 2 years of age females were much larger than males (104.0 ± 7.23 g, *n* = 14 and 45.75 ± 4.07 g, *n* = 9, respectively). Larval temperature did not have a significant effect on post-metamorphic body mass across the studied life stages (*F*_1,53_ = 1.18, *p* = 0.283).

**Figure 1.**
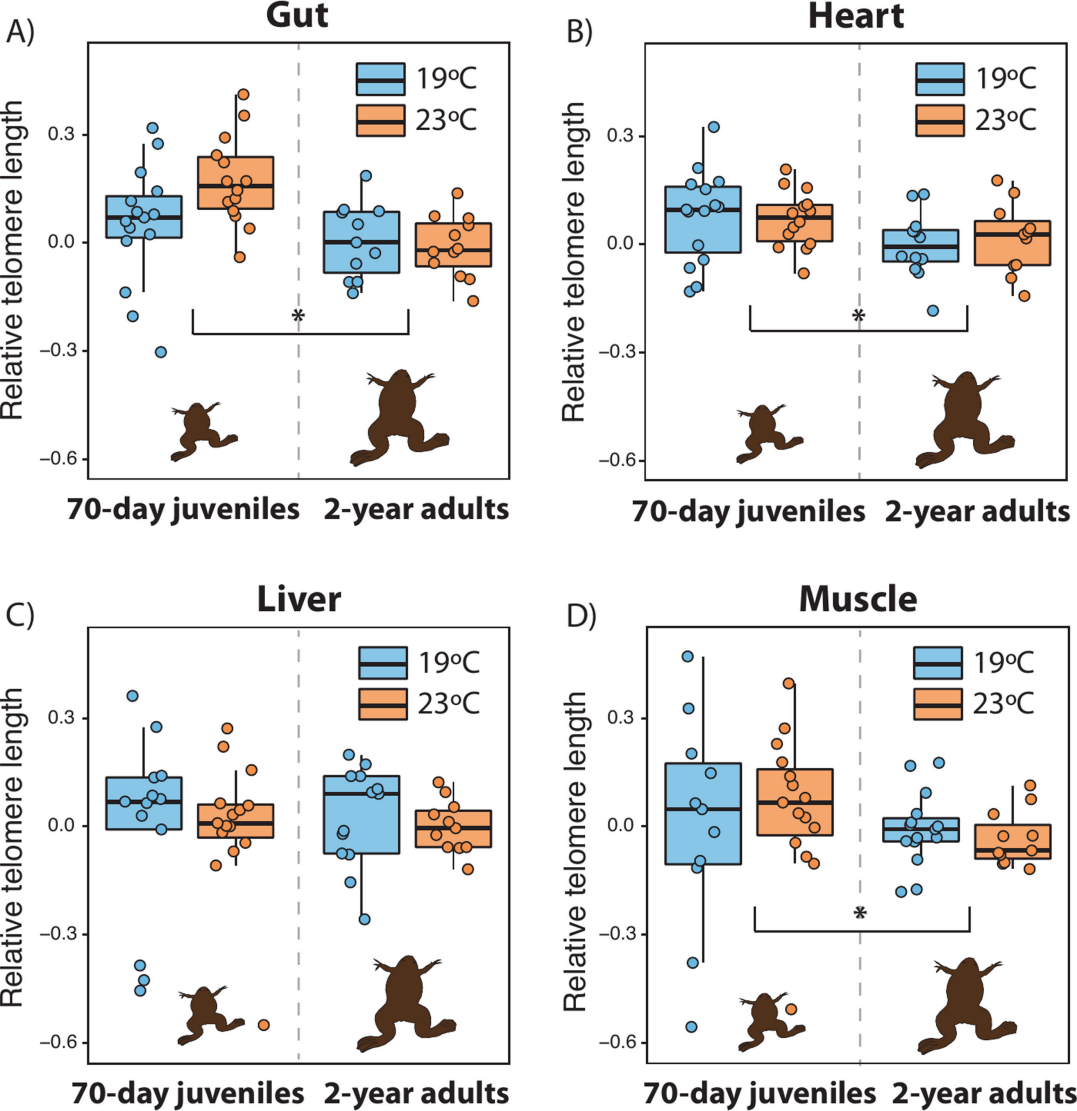
Variation in relative telomere length (log-transformed) in the (A) gut, (B) heart, (C) liver and (D) muscle of 70-day-old post-metamorphic juveniles and 2-year-old adults of *Xenopus laevis* that had been reared during the larval period at either 19°C (in blue) or 23°C (in orange). Boxes represent 25th to 75th percentiles, lines within boxes represent median values and vertical lines represent maximum and minimum data values. Lines with asterisks indicate significant differences in telomere length between juveniles and adults. Warmer temperatures at the larval stage resulted in shorter liver telomeres regardless of post-metamorphic life stage and greater reductions in gut telomere length from juvenile to adult (table 1). Drawings are not scaled.

**Figure 2.**
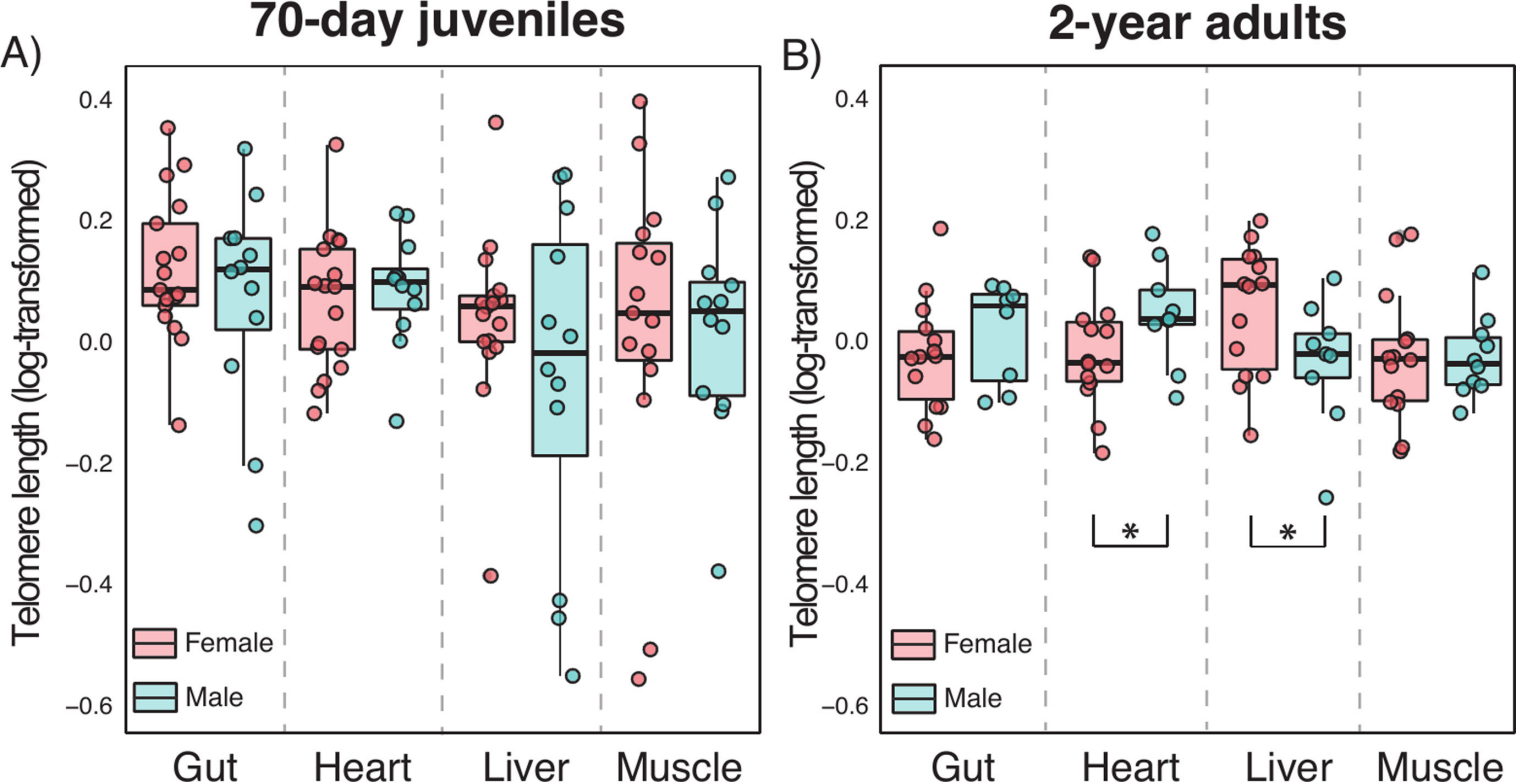
Telomere dynamics across organs and between sexes in (A) 70-day-old juveniles and (B) 2-year-old adults in *Xenopus laevis*. Boxes represent 25th to 75th percentiles, lines within boxes represent median values and vertical lines represent maximum and minimum data values. Lines with asterisks indicate significant differences in telomere length between females and males.

Changes in mean telomere lengths from the 70 day juveniles to 2-year-old adults varied across organs (figure 1; electronic supplementary material, table S1). In all the organs except the liver, telomeres were shorter in 2-year-old adult frogs than in 70 day juveniles (all *life stage* effect *p* < 0.038; figure 1; table 1). The temperature by life-stage interaction was significant only in the gut, with warm conditions at the larval stage being associated with longer telomeres at 70 days but not at 2 years (figure 1; table 1). Warm temperatures prior to metamorphosis had a negative overall effect on post-metamorphic liver telomere lengths in both juveniles and adults (figure 1 and table 1). We did not find any interaction between telomere length and sex or body mass in 70 day juveniles (figure 2, electronic supplementary material, figure S2). However, by the time that the frogs were 2-year-old adults, there was a significant organ-by-sex interaction (figure 2; electronic supplementary material, table S4), with heart and liver telomeres being shorter and longer in females than males, respectively (*post hoc p* = 0.031 and 0.047; figure 2). Gut and muscle telomere lengths did not significantly differ between sexes (figure 2; electronic supplementary material, table S4). In 2-year-old adults, there was also a marginally non-significant body mass by sex interaction, mostly caused by positive correlations between telomere length and body mass in males, but negative correlations in females (electronic supplementary material, figure S2; table S4).

**Table 1.** Linear mixed models testing for the effect of life stage (post-metamorphic 70-day-old juvenile versus 2-year-old adult), temperature during the larval period and their interaction on relative telomere lengths of the gut, heart, liver and muscle in *Xenopus laevis*. The variance explained by the fixed effects, and fixed plus random effects, is indicated by *R*^2^_m_ and *R*^2^_c_, respectively. Sample sizes for gut, heart, liver and muscle telomeres are, respectively, *n* = 30, 29, 30 and 30 for juveniles and *n* = 22, 23, 23 and 23 for adults. qPCR, quantitative polymerase chain reaction. Significant effects (i.e. *p-*values lower than 0.05) are shown in bold font.

	gut			heart			liver			muscle		
	*R*^2^_m_ = 0.26, *R*^2^_c_ = 0.32			*R*^2^_m_ = 0.10, *R*^2^_c_ = 0.10			*R*^2^_m_ = 0.07, *R*^2^_c_ = 0.63			*R*^2^_m_ = 0.04, *R*^2^_c_ = 0.62		
	d.f.	*χ* ^2^	*p*	d.f.	*χ* ^2^	*p*	d.f.	*χ* ^2^	*p*	d.f.	*χ* ^2^	*p*
*life stage*	1	12.18	**<0.001**	1	5.54	**0.019**	1	1.75	0.186	1	4.29	**0.038**
*temperature*	1	2.34	0.126	1	0.01	0.903	1	4.01	**0.045**	1	0.00	0.954
*life stage × temperature*	1	3.88	**0.049**	1	0.22	0.638	1	0.76	0.383	1	0.96	0.328
*qPCR set (random*)	1	0.147	0.701	1	0.01	0.999	1	29.4	**<0.001**	1	11.3	**<0.001**

**Figure 3.**
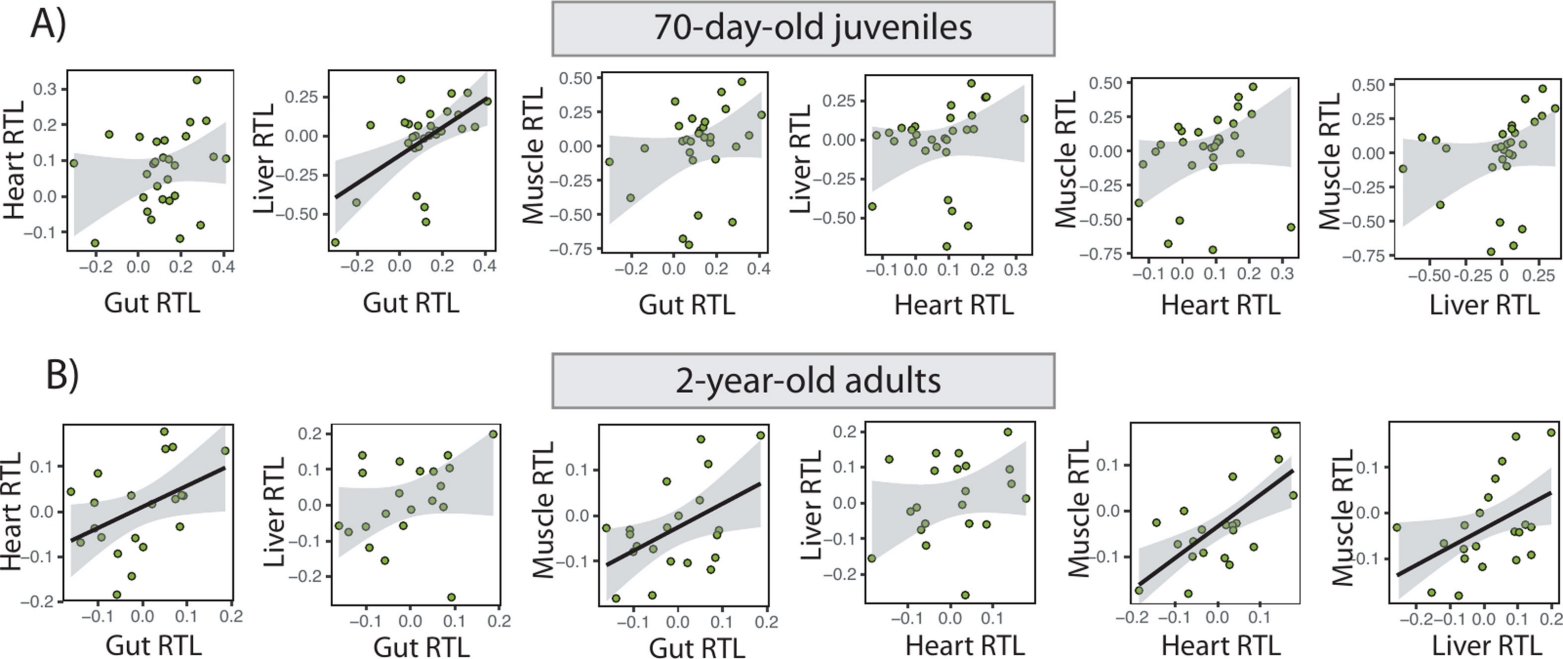
Linear correlations between (log-transformed) gut, liver, heart and muscle telomere lengths within the same individual in (A) 70-day-old and (B) 2-year-old *Xenopus laevis* frogs. Regression lines are shown when the linear correlation is significant.

We found positive Pearson correlations between telomere lengths in all the pairings of organs within an individual, whether juvenile or adult (figure 3; electronic supplementary material, table S2). In juveniles, only liver and gut telomere lengths were significantly correlated within an individual (figure 3; electronic supplementary material, table S2). However, the correlations between muscle–gut, muscle–heart and muscle–liver telomere lengths were significant in the adults and heart–gut was marginally non-significant (corrected *p*‐value = 0.06; figure 3; electronic supplementary material, table S2). Finally, adult liver telomere length negatively correlated with liver mass (once corrected for body mass), whereas the relationship between organ telomere length and organ mass was not significant for the other organs (electronic supplementary material, table S3).

## 4. Discussion

Here, we examined changes in telomere length in *X. laevis* in four body organs between 70-day-old post-metamorphic juveniles and 2-year-old adults. Telomere lengths in the cells of the gut, heart muscle and hindlimb muscle shortened across these life stages, whereas mean liver telomere lengths remained unaltered. While the data were cross-sectional, these patterns are unlikely to be driven by selective disappearance since mortality between 70 days and 2 years was relatively low (approx. 25%) and different organs show different trends for telomere length. As expected from the hypothesized relationship between temperature and ageing [27], warm (23°C) conditions during the larval period overall resulted in shorter telomeres in the liver (regardless of life stage). However, while we had observed longer telomeres in the gut of juveniles that had been exposed to warm temperature as larvae, this difference had disappeared in the adults.

Telomere dynamics are known to be shaped by a number of different processes. The enzyme telomerase is responsible for the maintenance of telomeres and, when this enzyme is not expressed, telomeres are thought to shorten with each cell division [46]. Telomerase expression is not common after embryogenesis in the somatic tissues of many endotherms (although this pattern varies with body size in mammals; [47]), which can partially explain telomere shortening after birth in these taxa [48]. In contrast, telomerase activity has been detected throughout the lifetime of some ectothermic animals, particularly those with indeterminate growth, which could be behind the weak or absent telomere shortening with age observed in some cases [49]. Also, despite the overall decrease often observed in telomerase expression and telomere length with age, their dynamics have been shown to be organ-specific and context-dependent in some species. For example, in fish (killifish and zebrafish), telomere length and telomerase activity increase from embryo to adulthood, and then both drastically decline in aged individuals, with telomeres shortening in the gut, kidney marrow and muscle but not in testis [11–13]. These patterns may indicate local and systemic ageing, and can be shaped by fish life histories [12,13]. In *X. laevis,* telomerase is detectable in adult somatic tissues studied here [50]; however, it is not known whether telomerase activity varies across life stages. In our study, we observed organ-specific telomere dynamics from juvenile to adulthood in frogs. We have previously shown that organismal growth, organ remodelling and environmental temperature during pre-metamorphic and metamorphic stages result in contrasting telomere dynamics across organs of *X. laevis* [9]. In the present study, the mean body mass of the frogs increased over 30-fold between the 70 day juvenile and 2 year adult sampling points, resulting in extensive organ growth. However, these organs do not all grow in the same way: the relative roles of hyperplasia and hypertrophy, as well as differences in cell turnover rate, may explain their different telomere dynamics. The mammalian gut seems to mainly grow through hyperplasia and experiences high cell turnover rates [51]; these two processes could explain shorter gut telomeres in adult frogs. In contrast, hypertrophy is common in heart and skeletal muscle, and both organs experience low cell turnover rates. Despite this, telomeres were shorter in adults than in juveniles in these two organs; hence, elevated production of free radicals may potentially have driven telomere shortening in these two organs [52,53]. Finally, the liver experiences great size increases due to hyperplasia, yet showed no change in mean telomere length over this time period. This may be a consequence of the high regeneration capacity of this organ, necessitating mechanisms (such as alternative telomere lengthening or the action of pathways providing protection from telomere attrition, e.g. antioxidants [53,54]) to buffer telomere attrition during development. Future research quantifying variation among organs in cell divisions (i.e. hyperplasia) and cell size (i.e. hypertrophia), together with the understanding of life stage-specific changes in mechanisms that can influence telomere length (e.g. telomerase activity, redox status, cell recruitment), will fully disentangle the mechanistic basis driving organ-specific telomere dynamics.

We also found evidence of sex-specific variation in telomere length across organs in adults, which may be influenced by differences in growth trajectories. Although current evidence does not suggest a role of sex in shaping adult telomere lengths across vertebrates, there is a lack of studies investigating telomere dynamics in taxa (such as amphibians) with marked sexual dimorphism [55]. Finally, we observed stronger correlations between telomere lengths of the different organs of an individual in adults than in juveniles, a pattern that may be driven by the organ-specific impact of metamorphosis on juvenile telomeres. Overall, these results show that, regardless of the mechanism behind organ growth, telomere shortening is observed with age across different organs in maturing frogs. This contrasts with what we previously observed at earlier post-metamorphic stages (seven-month-old frogs [9]), when individuals had attained much smaller body sizes (approx. 25 g on average at seven months old versus approx. 75 g at 2 years old).

The experimental manipulation of environmental temperature during the larval stage induced contrasting effects across organs and life stages. The warm early life treatment led to longer telomeres at the juvenile stage, but this effect had disappeared by 2 years of age. (It is important to note here that we did not find exactly the same pattern at 70 days in our previous study [9], possibly due to using different methods to extract DNA for the post-metamorphic samples used here [45].) Cellular processes involved in gut development, such as the migration of stem cells (that presumably have longer telomeres) and subsequent enhanced hyperplasia [56], might have taken place faster at higher temperatures. Likewise, hyperplasia might explain shorter telomeres in the liver of post-metamorphic frogs that experienced early-life warming. Studying the interplay between temperature, organ growth, cell turnover rate and telomere dynamics will help us to understand the mechanics of organ ageing in animals coping with challenging thermal scenarios.

## 5. Conclusions

This study confirms that telomere dynamics over the period from early to sexually maturing post-metamorphic frogs (i.e. 70 day versus 2 year individuals) differ between organs. However, our results contrast with the patterns previously observed across younger post-metamorphic life stages of the same species (up to seven months; [9]). This suggests that age and/or organ growth play a role in eroding amphibian telomeres over time, in a manner that differs across organs and also in response to early life conditions. Future research should ideally investigate whether telomere dynamics in post-metamorphic amphibians are linked to organismal fitness and survival (e.g. [57]) and how this differs between organs. This will be technically challenging, since it will require non-terminal sampling (i.e. biopsies) so that changes in telomere length can be measured over time within the same individual, with these changes then being related to subsequent fitness.

## Data Availability

Data supporting this article are available at [58]. Supplementary material is available online [59].
